# The Putty Index Technique for Anterior Tooth Fracture Restoration: A Case Report

**DOI:** 10.7759/cureus.67109

**Published:** 2024-08-18

**Authors:** Arshjot S Basra, Joyeeta Mahapatra

**Affiliations:** 1 Department of Conservative Dentistry and Endodontics, Sharad Pawar Dental College and Hospital, Datta Meghe Institute of Higher Education and Research, Wardha, IND

**Keywords:** putty rubber base impression material, anterior tooth fracture, composite restoration, anterior aesthetic restoration, putty index technique, ellis fracture

## Abstract

Traumatic injuries of anterior teeth can seriously affect a person's smile, which contributes significantly to an individual’s overall personality and sense of self. Hence, various efforts have been made over the years to develop techniques that enable clinicians to replicate the natural tooth anatomy while being practical enough to allow its use in clinical practice. The putty index technique helps to make significant progress in achieving such goals by forming a template against which composite material can be placed for restoration of various modalities - in this case, Ellis class I and II. The prepared index replicates the palatal anatomy of the wax build-up done on the patient's cast. This reduces the chair time while improving the predictability of the final restoration.

## Introduction

In clinical practice, dentists frequently come across cases sustaining injuries in the anterior region of the jaw. These types of injuries are often linked to maxillary incisors due to their vulnerable setting in the arch, which increases their risk of fracture. These fractures can have a variety of causes, such as road traffic accidents, athletic injuries, and trauma from malocclusion brought on by crowding of the anterior teeth [1]. Traumatic injuries have been classified by various authors, among which the most common is the Ellis classification system [2]. It is a detailed system specifically designed to classify traumatic dental injuries of the anterior teeth such as incisors and canines.

Direct composite restorations offer minimally invasive and aesthetically pleasing results in restoring anterior teeth. However, achieving optimal anatomical form, accurate contact points, and a natural surface texture during freehand placement can be a significant challenge, especially for less experienced dentists. The putty index technique has emerged as a reliable and predictable method to address these concerns [3]. In this technique, the fabricated putty index acts as a valuable tool for dentists who seek to restore the anterior teeth with both precision and efficiency. It leverages a pre-fabricated silicone mold to guide the placement of composite resin, ensuring predictable and aesthetically pleasing results. The putty index technique involves the following stages: (A) impression and wax-up, (B) putty index fabrication, (C) tooth preparation and isolation, and (D) composite placement and finishing [4,5].

This report illustrates the clinical steps involved in employing the putty index technique for the restoration of an anterior tooth and highlights the aesthetic and functional outcomes achieved.

## Case presentation

A 27-year-old male patient reported to the Department of Conservative Dentistry and Endodontics, Sharad Pawar Dental College and Hospital, Wardha, India, with the chief complaint of a fractured tooth in the upper front region of the jaw. The patient had a history of a road traffic accident one year ago, which had led to a fracture in the upper front region of the jaw; there was no associated history of mobility, pain, or swelling. No significant medical history was present. On extraoral examination, the face was bilaterally symmetrical, the lips were competent, and the temporomandibular joint (TMJ) was bilaterally smooth and synchronous with no clicking sound.

**Figure 1:**
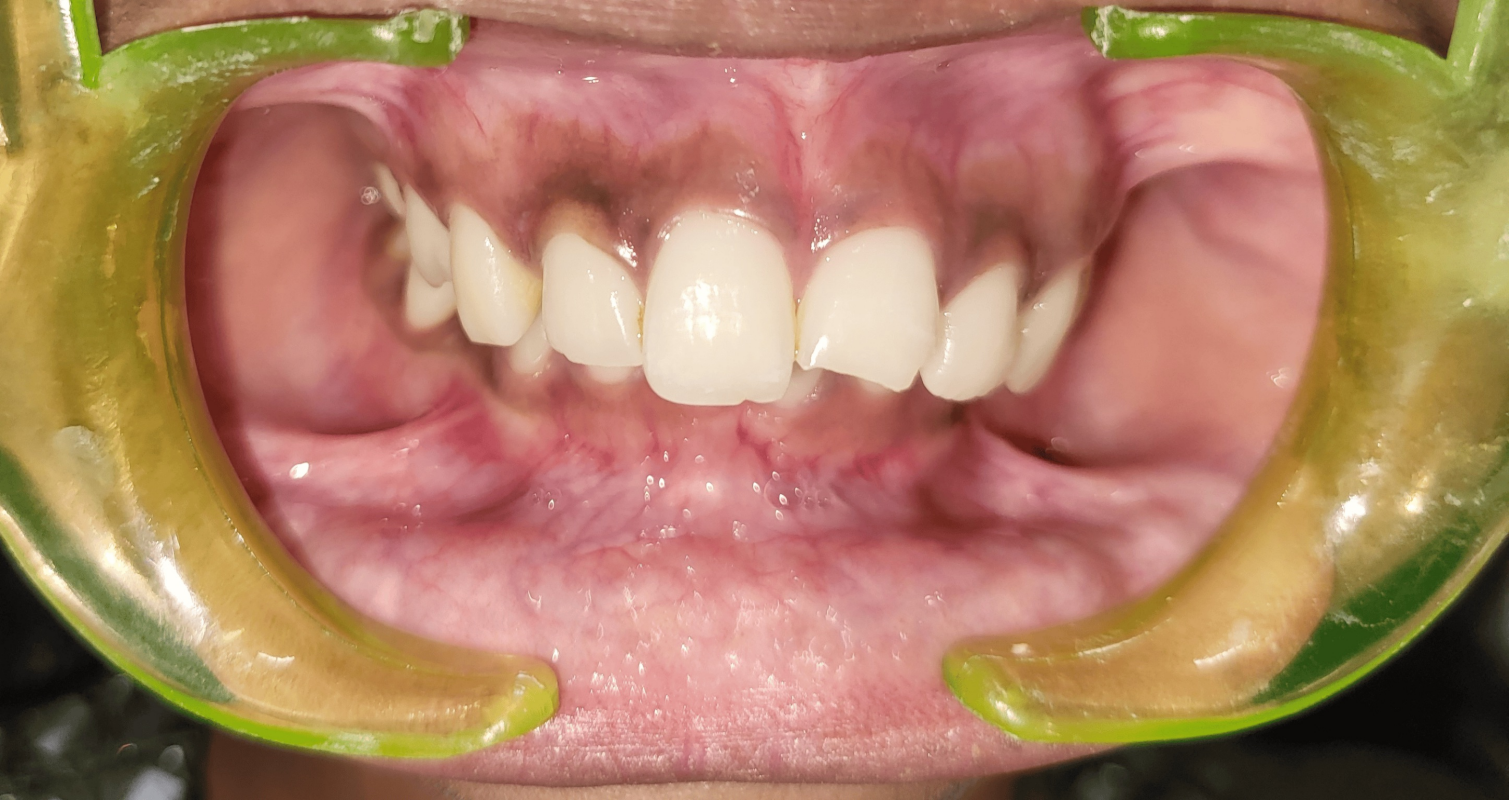
Intraoral preoperative profile view of the patient

**Figure 2:**
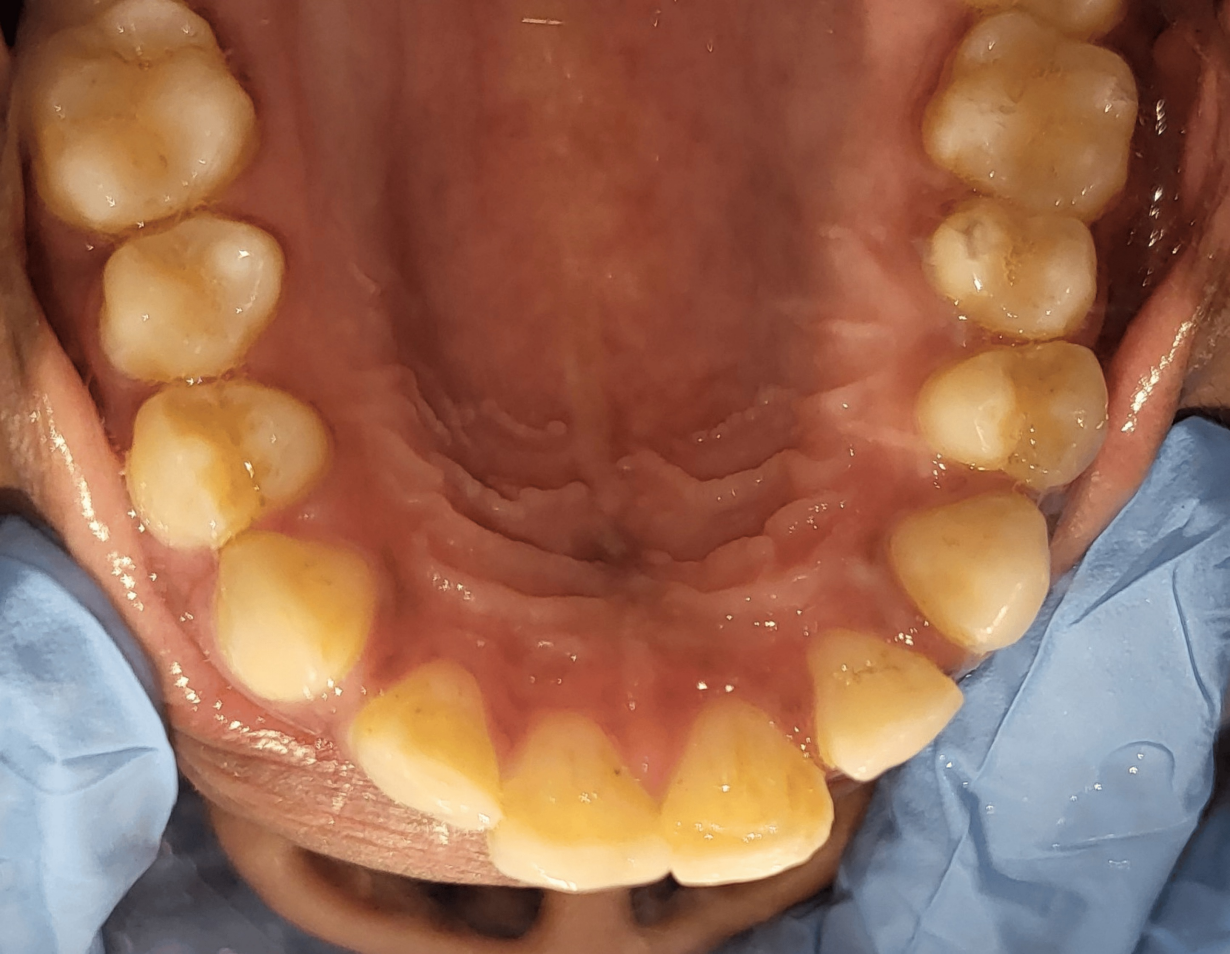
Occlusal view of the patient’s maxillary jaw

On intraoral examination, an Ellis class I fracture with tooth number 11 and an Ellis class II fracture with tooth number 21 were present (Figures 1-2). The neural sensibility test was done using electric pulp testing (Parkell Digitest II Pulp Vitality Tester, Parkell Inc., Brentwood, NY) and a cold test using Endo Ice (Coltene Vitality Control Endo-Frost, COLTENE Inc., Altstätten, Switzerland). Based on the tests mentioned above, it was inferred that both teeth were vital.

**Figure 3:**
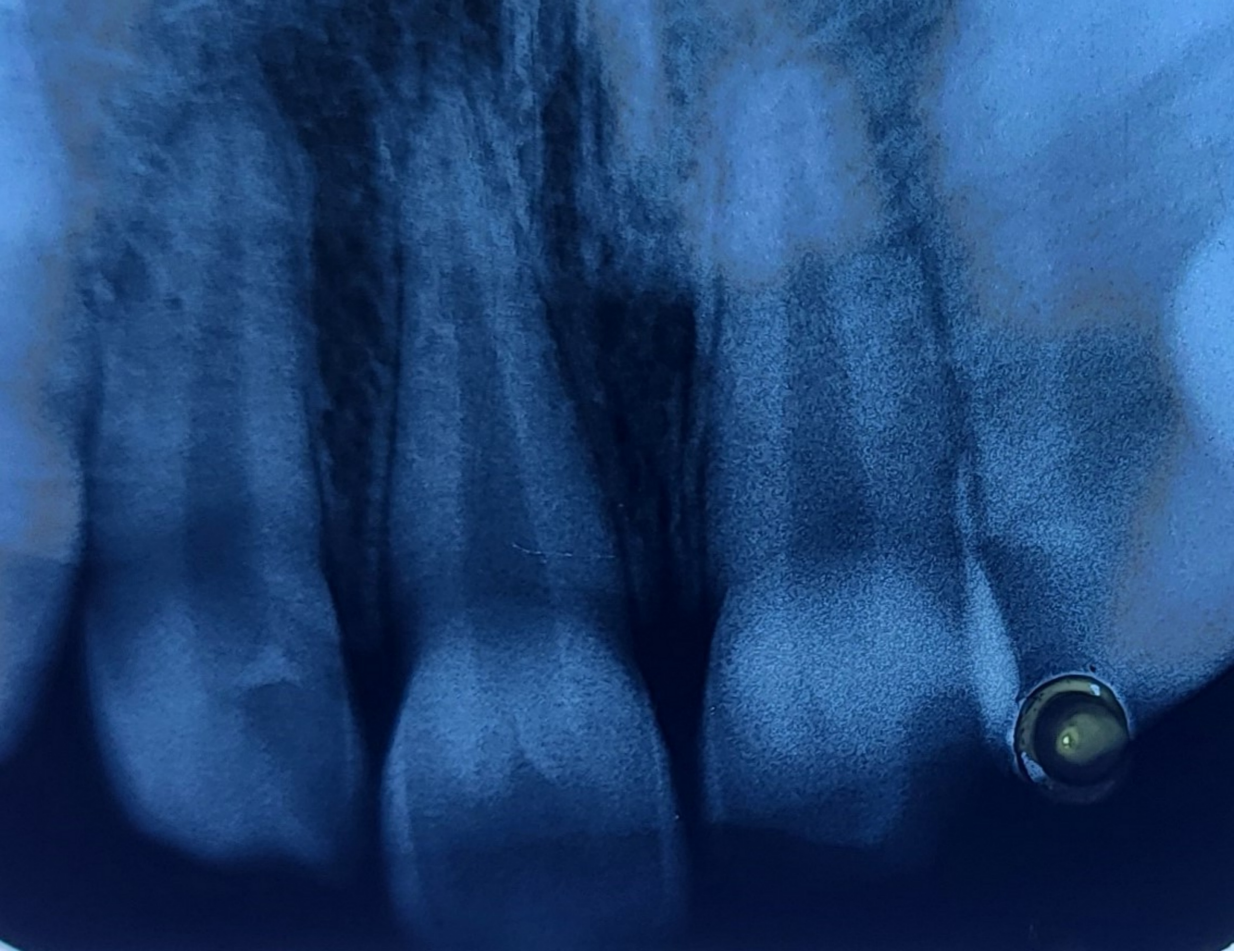
Intraoral periapical radiograph (IOPA) of tooth numbers 11 and 21

An intraoral periapical radiograph (IOPA) of tooth numbers 11 and 21 was taken (Figure 3). On the radiograph, radiolucency involving the enamel and dentin at the incisal third of tooth number 21 was seen, whereas, with tooth number 11, the fracture only involved enamel at the incisal third. Radiolucency did not involve the pulp chamber in either of the teeth and no periodontal ligament (PDL) space widening was visible in the apical area of tooth numbers 11 and 21.

A final diagnosis of asymptomatic reversible pulpitis with 11 and 21 was reached.

Treatment

**Figure 4:**
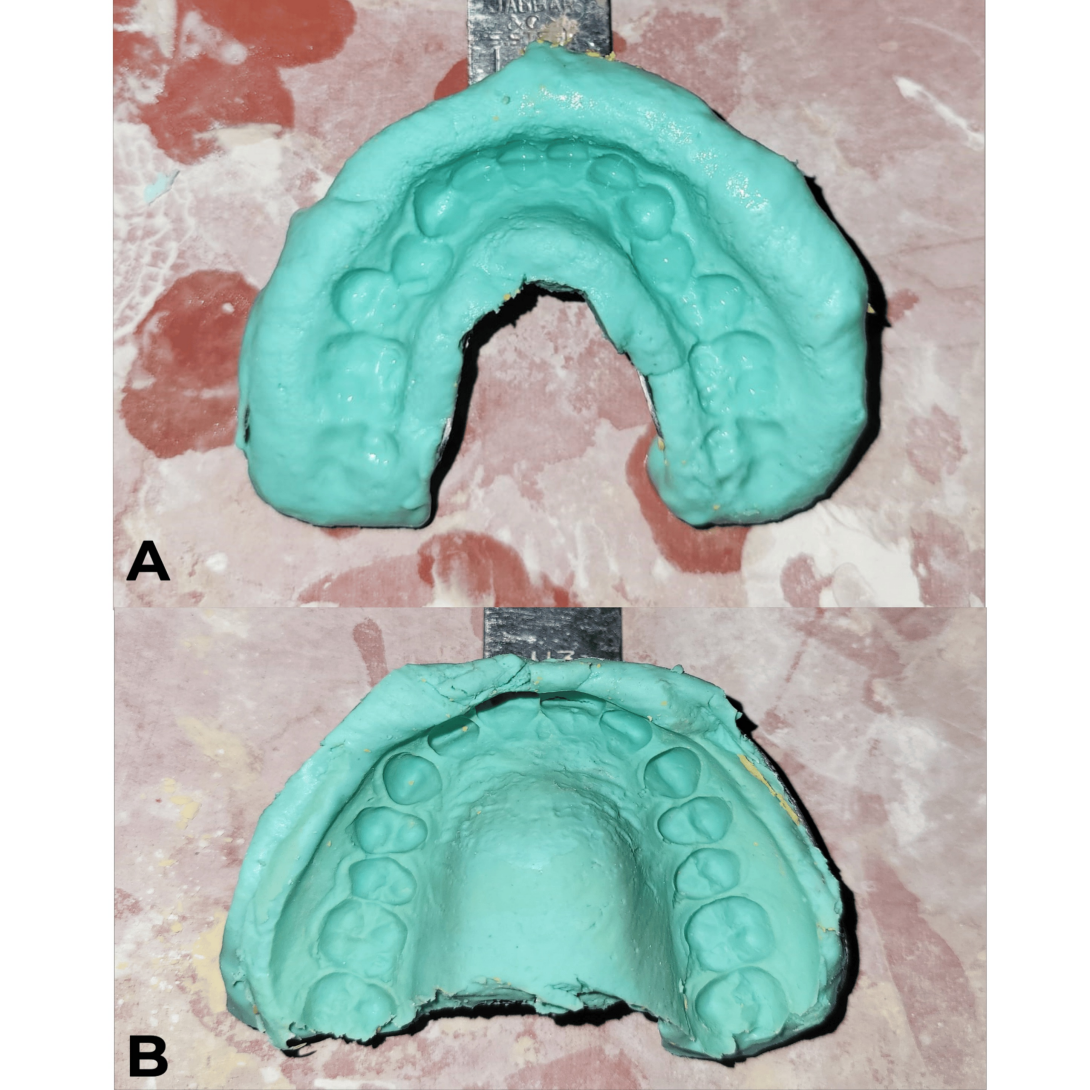
Alginate impression made on patient’s first visit A) Alginate impression of the patient’s mandible. B) Alginate impression of the patients maxilla

**Figure 5:**
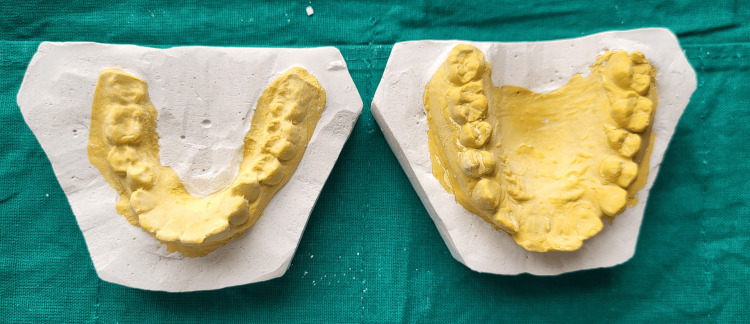
Occlusal view of maxillary and mandibular casts

**Figure 6:**
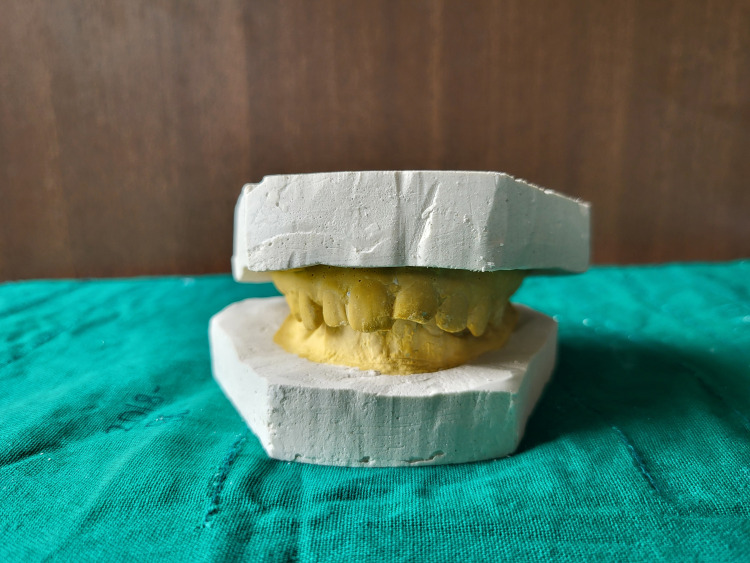
Profile view of maxillary and mandibular casts

**Figure 7:**
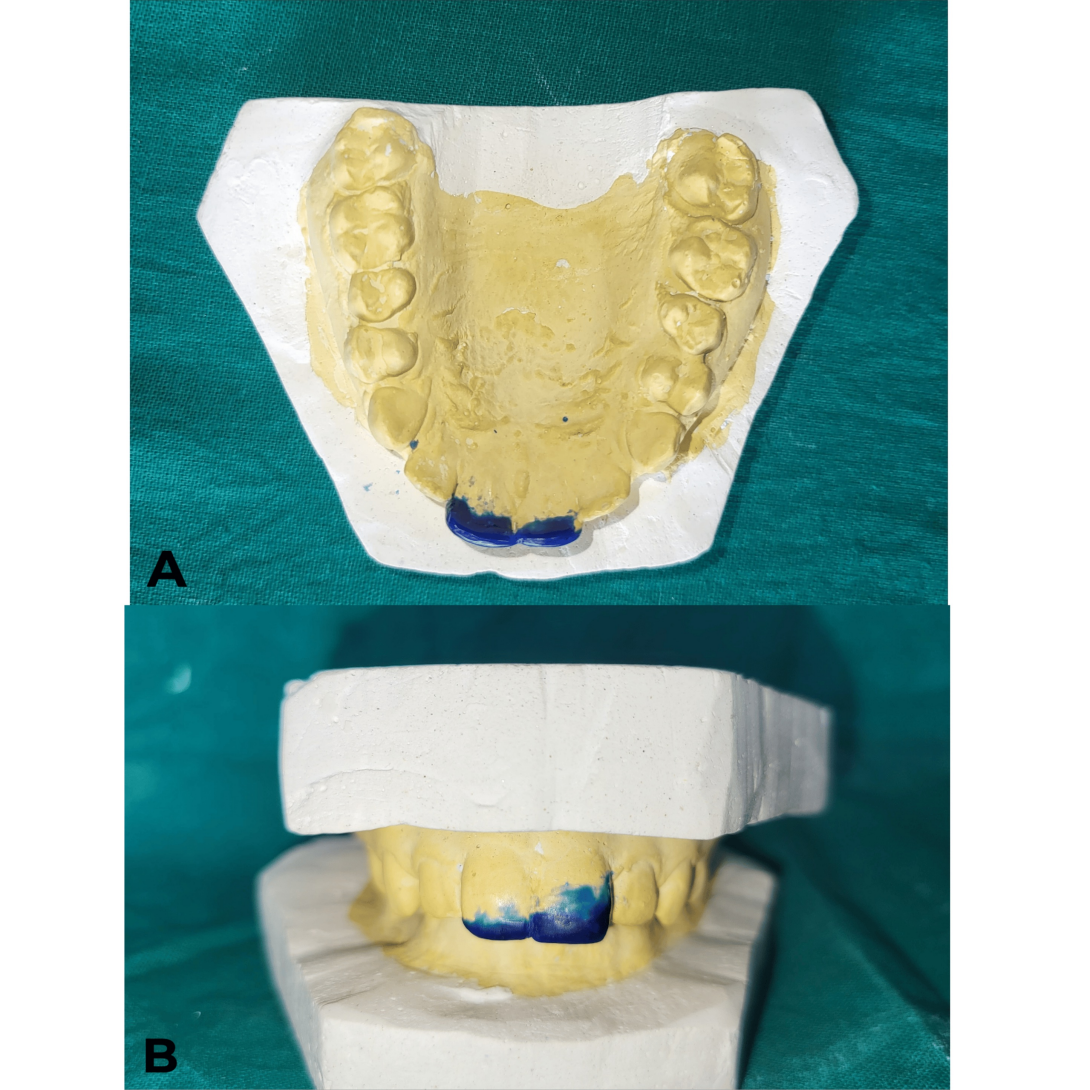
Wax build-up done with tooth numbers 11 and 21 A) Wax build-up on the maxillary arch in the occlusal view. B) Wax build-up profile view with the maxillary and mandibular arch in occlusion in the profile view

On the first visit, the procedure was explained to the patient, and written informed consent was obtained. Alginate impressions (Algingum alginate impression material, Prime Dental Products PVT LTD, Thane, India) of the patient’s maxillary and mandibular arch were made (Figure 4), which were later used to make diagnostic casts that replicated the patient's oral cavity (Figures 5-6), on which wax build-up was done (2GM Blue Inlay Wax, Giriraj Products, Mumbai, India), which in turn replicated the desired final restoration (Figure 7).

**Figure 8:**
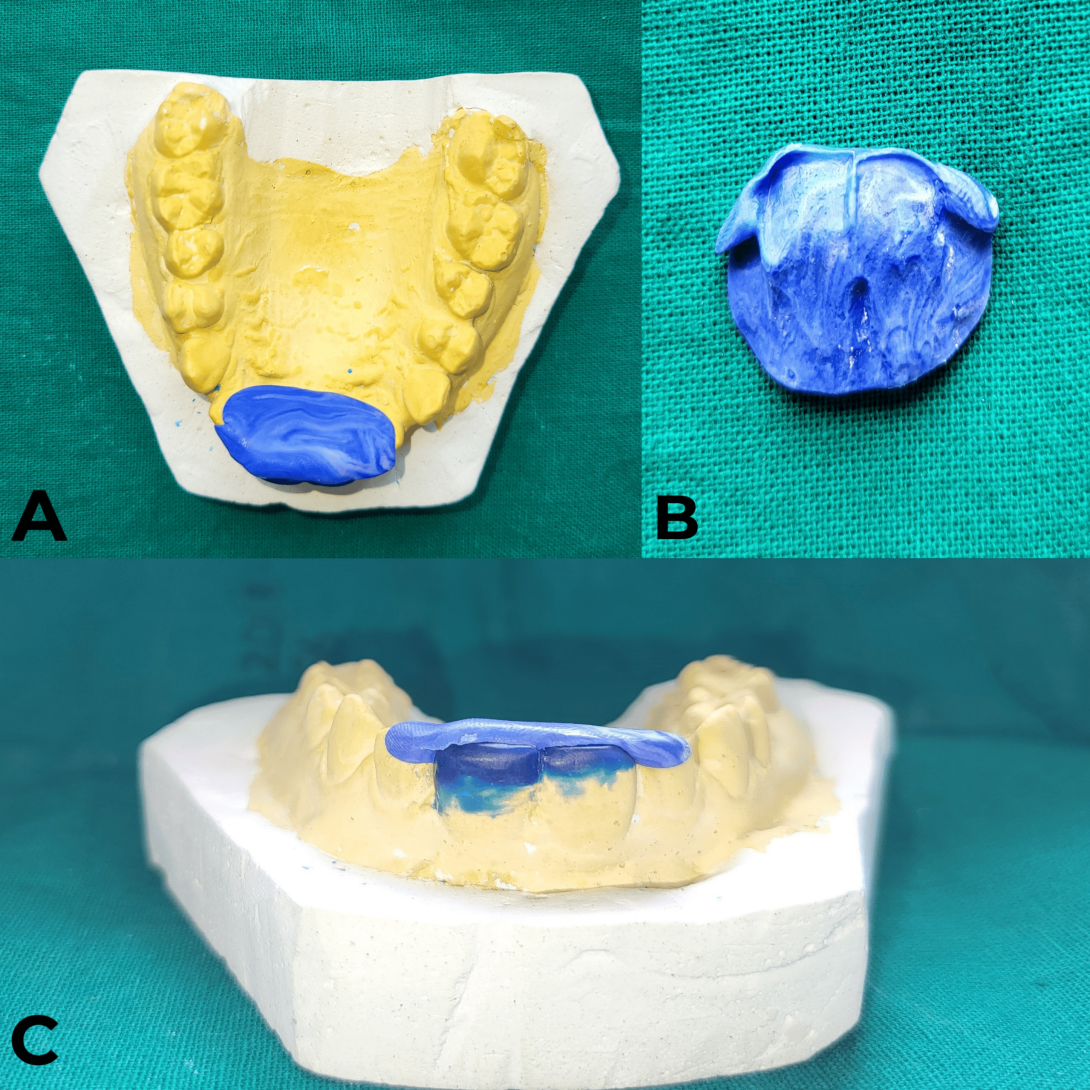
Prepared putty Index on tooth numbers 11 and 21 A) Prepared putty index on the maxillary cast in the occlusal view. B) Putty index replicating the palatal anatomy of the prepared tooth along with wax build-up. C) Putty index placed against the palatal surface of tooth numbers 11 and 21

Vinyl polysiloxane putty (Putty Normal Set, Zhermack Hydrorise, Badia Polesine, Italy) was used to create an index from the prepared cast by fabricating an impression of wax build-up (Figure 8); this served as a template for shaping the composite restoration during the clinical procedure.

**Figure 9:**
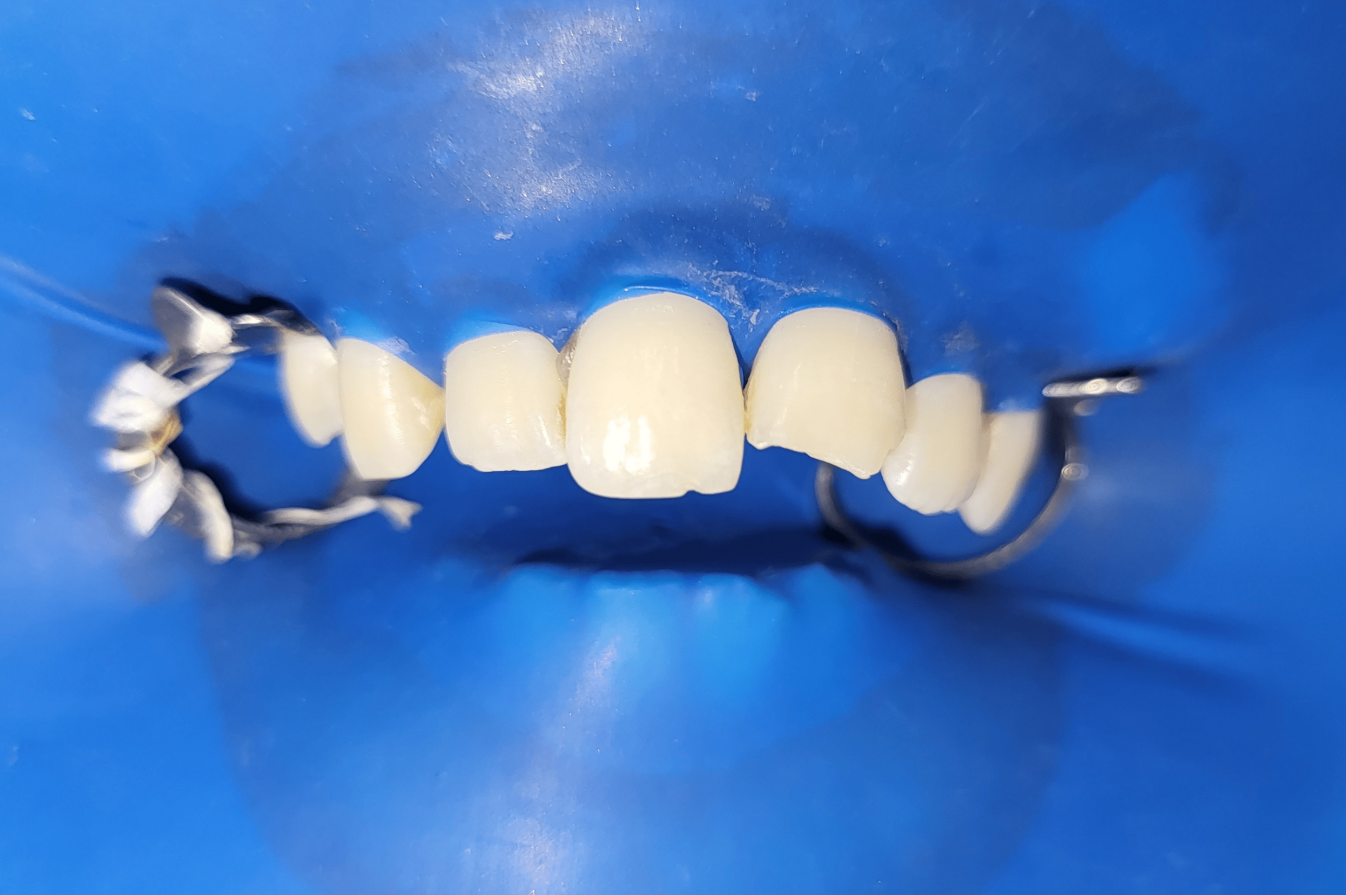
Rubber dam isolation done on tooth numbers 13, 12, 11, 21, 22, and 23

**Figure 10:**
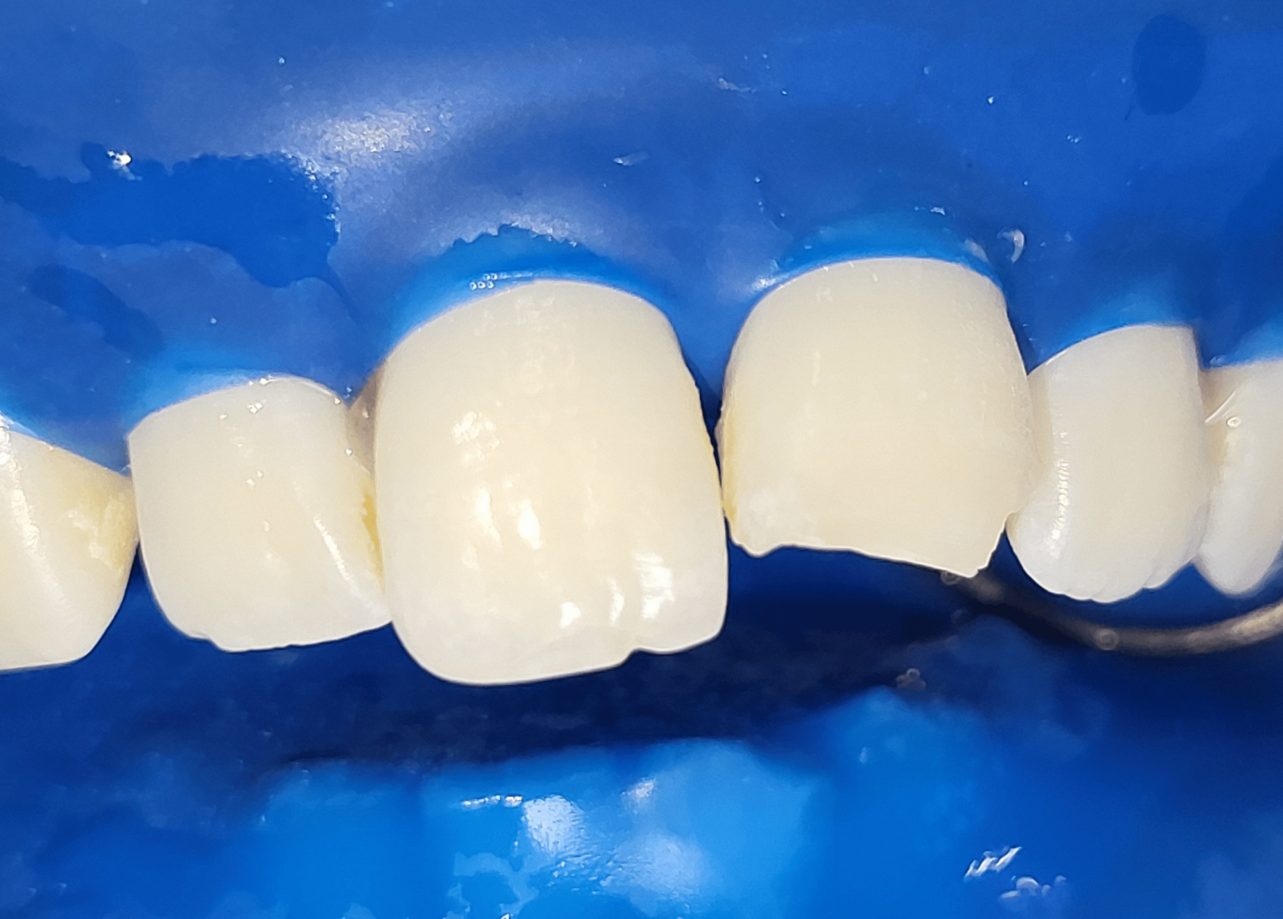
Beveled tooth surface of tooth numbers 11 and 21

On the second visit, the shade-matching of the composite for restoration was done by placing the composite buttons on the unprepared tooth surface and clinically observing the best shade for restoration, followed by rubber dam isolation for tooth numbers 13, 14, 12, 11, 21, 22, 23, and 24 (Figure 9). Once the shade was selected (A2 composite shade Dentsply Spectrum® Universal Micro Hybrid Composite, Bensheim, Germany), beveling of the tooth surface was done using TF-12 bur (Mani® diamond bur, Utsunomiya, Japan) (Figure 10).

**Figure 11:**
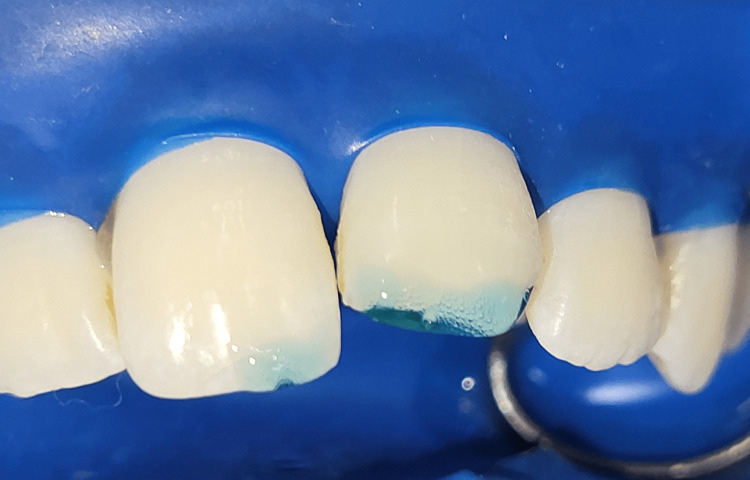
Etching of the prepared tooth surface with 37% phosphoric acid

**Figure 12:**
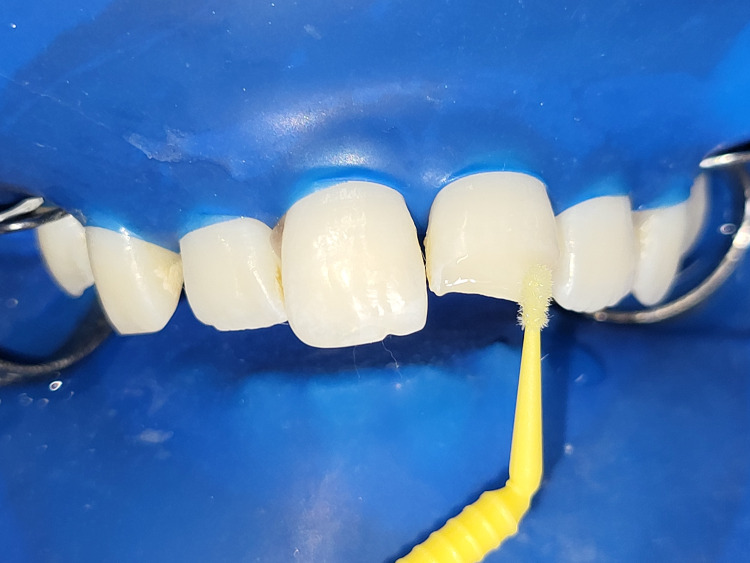
Bonding agent application onto the etched tooth surface by applicator tip

**Figure 13:**
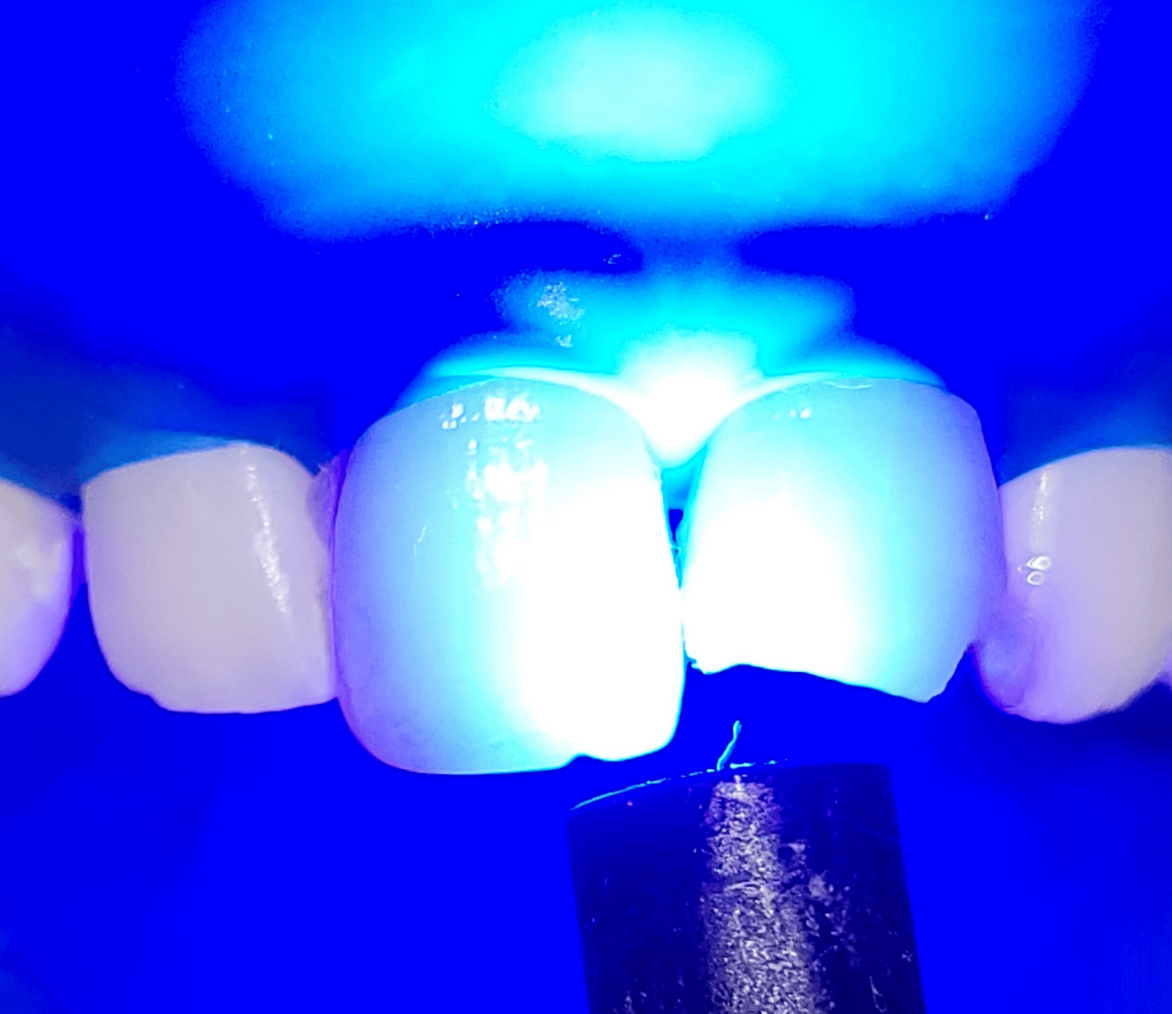
Light-curing of bonding agent

Etching (37% phosphoric acid, Prime Dental Products Pvt. Ltd., Thane, India) was done for 15 seconds (Figure 11), followed by rinsing the tooth surface with water. Later, the bonding agent (3M ESPE Adper Single Bond 2, 3M India Ltd, Bangalore, India) application onto the prepared tooth surface was done by the applicator tip in two layers, which were later light-cured for 20 seconds (Figures 12-13).

**Figure 14:**
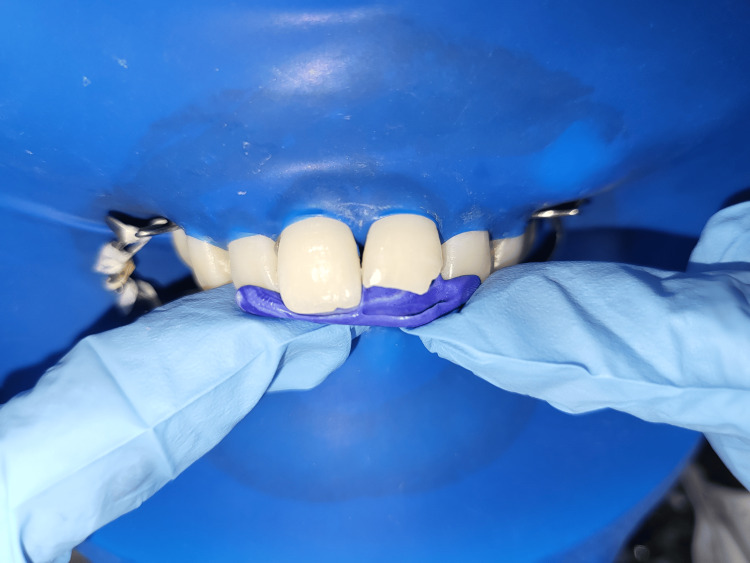
Putty index placed on the palatal surface of tooth numbers 11 and 21

**Figure 15:**
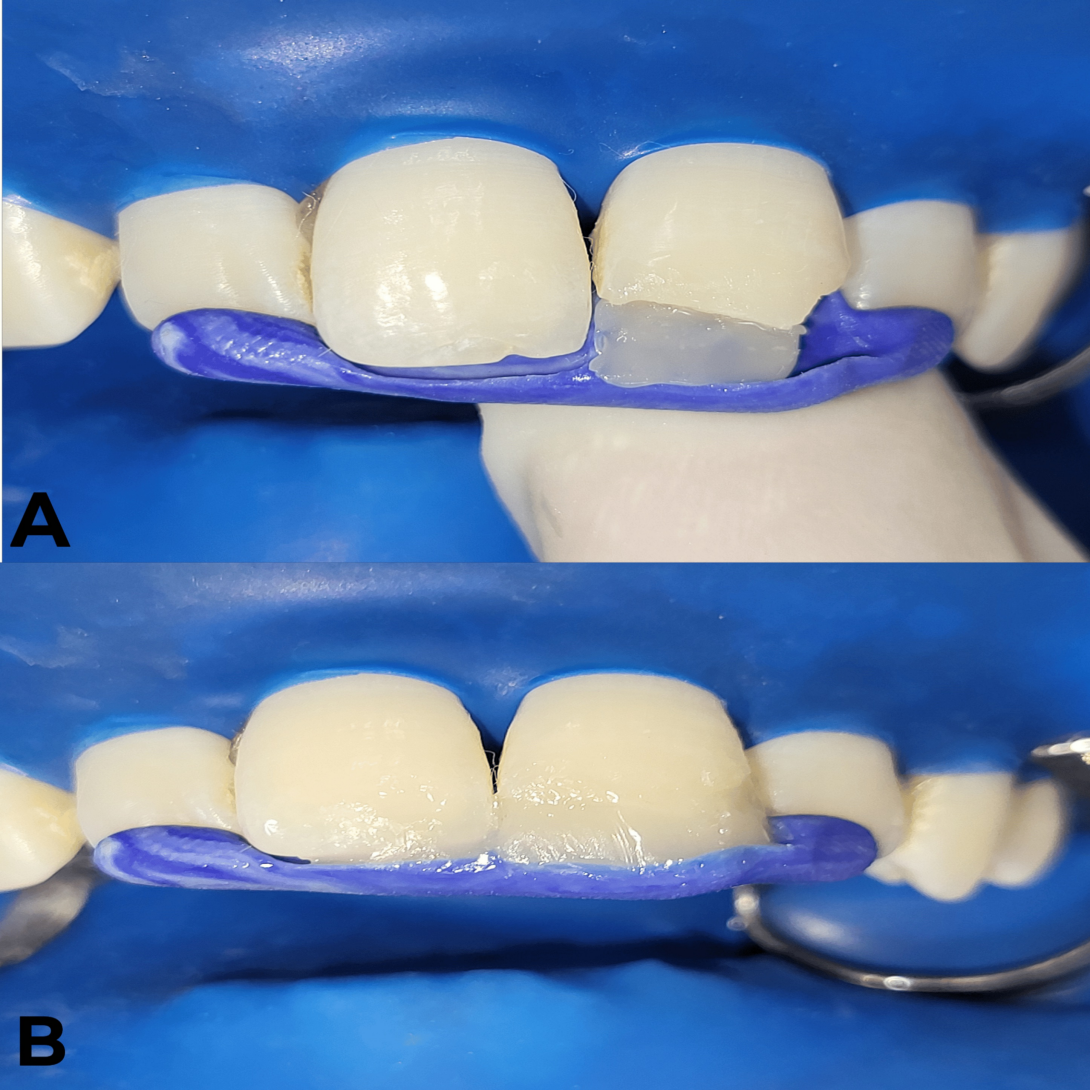
Incremental placement of resin composite restoration against the prepared index with tooth numbers 21 and 11 A) Composite restoration placed against the index with tooth number 21. B) Composite restoration placed against the index with tooth numbers 21 and 11

The prepared index was placed against the palatal aspect of tooth numbers 11 and 21 (Figure 14), and resin composite material was added incrementally against the index. Once the build-up was done, final curing was undertaken for 40 seconds (Figure 15). For finishing and polishing, extra-fine tapered diamond TF-11 bur (Mani® diamond bur, Utsunomiya, Japan) was employed for the coarse finishing of the composite. Final finishing and polishing were done with a series of abrasive disks (Shofu Super Snap mini kit, Kyoto, Japan).

**Figure 16:**
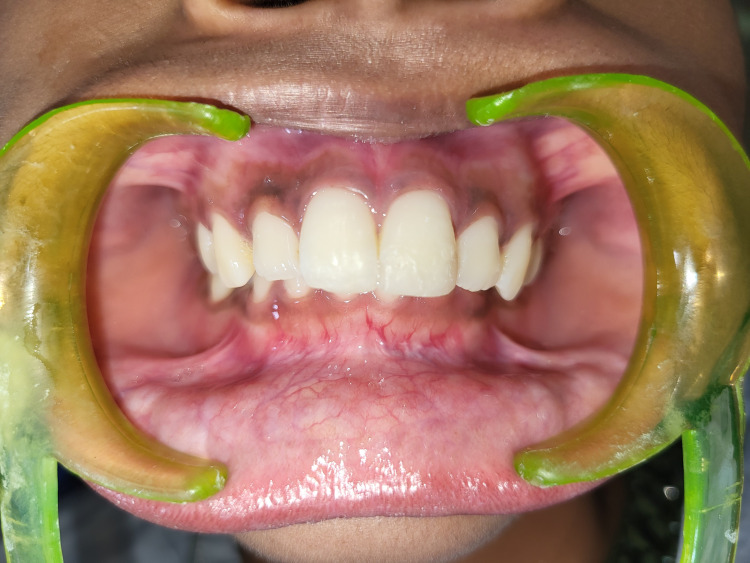
Postoperative profile view of the patient's oral cavity

**Figure 17:**
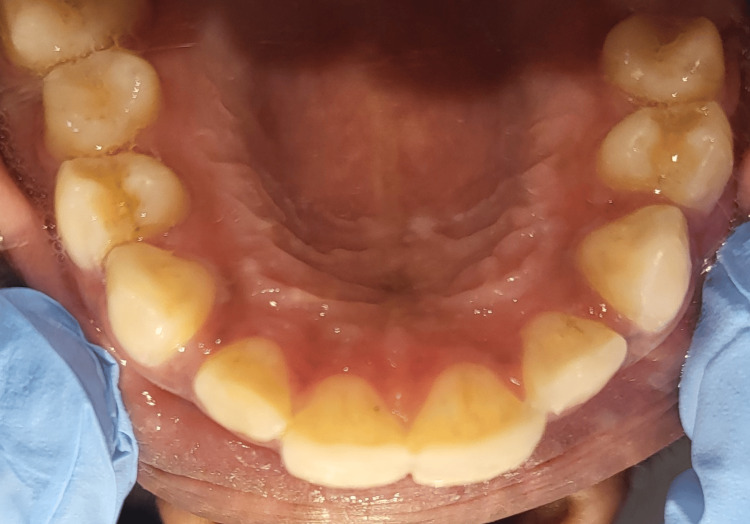
Intraoral postoperative occlusal view of the patient

Postoperatively, the fracture line was no longer visible (Figures 16-17), and the patient was satisfied with the results.

## Discussion

Trauma to the anterior teeth can lead to aesthetic, phonetic, and functional complications. A common consequence of injuries to the anterior teeth is crown fractures, which may or may not affect the dental pulp. Fractures that do not involve the tooth pulp (Ellis class I and II) (Table 1) can be easily managed by using direct composite resin by employing techniques like putty index and strip crown technique [6].

**Table 1:** Ellis and Davey classification of tooth fracture Table Credit: Arshjot S. Basra

Class	Description
I	Enamel fracture
II	Enamel and dentin fracture (pulp not involved)
III	Enamel, dentin, and pulp fracture
IV	Non-vital tooth (previous trauma)
V	Root fracture
VI	Fracture with tooth displacement
VII	Displacement of the tooth without fracture
VIII	Fracture of the crown en masse (complete crown fracture)
IX	Fracture of deciduous (primary) tooth

Since composites are one of the most commonly used materials for the rehabilitation of anterior teeth abnormalities, a physician must possess significant expertise regarding the usage of this material [7]. Due to its various benefits, which include immediate restoration, aesthetics, minimal invasion, cost-effectiveness, adhesion to the tooth structure, and reduced chairside time, direct anterior composite restoration has become increasingly popular. In the case of diastema closure, porcelain veneers, crowns, and Ellis class I and class II fractures, direct adhesive restorations are frequently performed for the treatment of uncomplicated crown fractures [8]. However, the success rates of direct composite restorations in permanent anterior teeth can vary, as described in the systemic review by Heintze et al. [9].

With direct composite restorative materials, the putty index restores the shape and functionality of the teeth while replicating the palatal contour. It also acts as a template to hold the restorative material, analyze the teeth's cervico-incisal length, and thickness, and permits the clinicians to meticulously preplan the procedure in advance (Table 2) (Video 1) [10-12].

**Table 2:** Putty index technique: process Table Credit: Arshjot S. Basra

Stage	Description
Impression and wax-up	Upper and lower arch impressions are made, casts are created, and a wax build-up is done to replicate the desired final restoration [1,4]
Putty index fabrication	A mold (putty index) is made from the cast using polyvinyl siloxane to serve as a guide for shaping the composite restoration [4]
Tooth preparation and isolation	Minimal enamel is removed from the affected tooth, followed by standard isolation procedures [3]
Composite placement and finishing	The putty index is used to place composite resin in increments, ensuring accurate anatomy. The composite is then cured, and polished for aesthetics and optimal function [5]

**Video 1:** Illustration of the putty index technique Video Credit: Arshjot S. Basra

The putty index technique offers several advantages over freehand methods, as evidenced by existing literature (Table 3). However, its disadvantages include the need for a mock-up build-up, the likelihood of a second appointment, and the possibility of the composite build-up adhering to the adjacent tooth, particularly if it is not isolated.

**Table 3:** Advantages of the putty index technique Table Credit: Arshjot S. Basra

Advantage	Description
Enhanced predictability	The pre-fabricated index ensures accurate replication of the desired tooth anatomy, reducing the need for extensive intraoperative adjustments and potential inaccuracies [1]
Improved efficiency	Streamlining the composite placement process by utilizing the index as a guide can potentially reduce chair time [3]
Simplified contouring	The index acts as a template for shaping the composite, leading to more consistent and predictable results, especially for less experienced practitioners [4]
Potential for improved aesthetics	By ensuring proper anatomical form and facilitating a natural surface texture, the putty index technique can contribute to a more aesthetically pleasing restoration [5]

Several authors have described methods for placing composite restorations in anterior teeth using either putty index alone or putty index in conjunction with a flexible matrix (PTFE Teflon tape and mylar strip) [7]. A case report by Sherwood et al. explores a technique where once acid etching and bonding agent application of the tooth to be restored is done, the conventional technique is modified by placing a mylar strip onto the adjacent tooth to stop the composite material from sticking to the tooth surface. This technique combines both rigid and flexible matrices, leading to the desired labial and palatal contour and finish [7]. Another study by Siddiqui et al. compared the putty index technique and the custom template technique in which a clear biocryl sheet measuring 0.75 mm was used to make the custom template [4]. It has been employed for the management of patients with amelogenesis imperfecta for full-mouth rehabilitation [13]. The custom template provides three-dimensional control over the contours while restoring multiple teeth at once and reduces the need for a clinician's expertise. However, no statistically significant difference was observed when custom template and putty index techniques were compared on key parameters such as anatomical form and clinical assessment [4,14]. Nevertheless, the custom template method did perform better on factors like secondary caries, color stability, and surface roughness in recent studies [4,14].

## Conclusions

Traumatic injuries of the face contribute significantly to cases of tooth fracture, which very often affects the patient’s appearance. Efforts have been made to manage such cases with minimally invasive techniques whenever indicated. The putty index technique enables clinicians to restore the affected tooth with proper palatal contour and plan the restoration beforehand on the prepared cast, which eventually results in improved aesthetics as compared to direct restoration by the freehand method where giving ideal palatal contour can be a challenge. Various efforts have been made to further extend the practicality, usability, and convenience offered by this technique, including the use of mylar strip and a custom template. This modification ultimately strives to improve the quality of treatment provided by the clinician.
